# Evaluation of the corrosion resistance of bronze patina or/and protective coating on the surface of the archaeological coins

**DOI:** 10.1038/s41598-025-85290-x

**Published:** 2025-01-18

**Authors:** Saleh M. Saleh, Abd El-Hakim A. El-Badry, Amal M. Abdel-karim

**Affiliations:** 1 Restoration and Conservation of Artifacts Department, Faculty of Archaeology, Fayoum University, Fayoum, Egypt; 2 Conservation Laboratory, ARCE, Karnak Temples, Luxor, Egypt; 3 Physical Chemistry Department, National Research Centre, 33 El Bohouth Street, P.O.12622, Dokki, Giza, Egypt

**Keywords:** Bronze coin-fragments, Bronze disease, Patina, Paraloid B-72® coating, Electrochemical measurements, Conservation, Electrochemistry, Physical chemistry

## Abstract

Archaeological coins are considered essential sources of historical documentation. Over time, they are subjected to corrosion processes that gradually alter their appearance, shape, and composition. This study aims to evaluate the effects of the patina and/or protective coating on the corrosion process. Protection of the original coin surface was crucial following the completion of the cleaning protocol. Various finishes of coin fragments (uncoated, aged, and freshly coated) were investigated to determine their chemical composition, nature of the patina, and corrosion products on their surface using stereo microscopy(SM), X-ray diffraction (XRD), and scanning electron microscopy (SEM) equipped with energy dispersive X-ray spectroscopy (EDX). The analysis revealed that the coins were composed of a Cu–Sn- and Pb bronze alloy. Furthermore, the efficiency of the patina and/or protective coatings on the coin fragments was evaluated using potentiodynamic polarization (PDP), electrochemical impedance spectroscopy (EIS), and cyclic voltammetry (CV) techniques. The highest protection was achieved for patinated-freshly protective coated fragments, while the most corrosive fragments were those affected by bronze diseases.

## Introduction

Archaeological metal artifacts are often completely or partially covered by corrosion products. Corrosion reactions are the mainly hazardous phenomena affecting metal heritage in various environments, including the indoor environment of museum, outdoor atmosphere^1,2^, and burial soil^3–5^.

Coins are small objects with intrinsic value, containing information related to architecture, culture, and arts of their time. They often bear images of rulers, national or religious symbols, and inscriptions, offering insights into the political and economic history of their period. Many coins show important buildings, temples, and other architectural marvels, serving as small canvases for the artistic styles of their period. The study of coins combines elements of history, archaeology, and art history^6–8^.

Bronze represents a class of alloys widely used in ancient times, valued for its hardness and greater corrosion-resistant compared to pure metals. Ancient cast bronze typically contains copper with varying amounts of tin, and zinc depending on the desired properties of the alloy^9,10^. The addition of lead (Pb) improved the fluidity of molten bronze, making it a preferred material for production of decorative coins^11–13^, particularly during the Greco-Roman ages^14^. The corrosion products of bronze are mainly composed of copper oxides, forming a uniform pale brown to black-brown layer known as the primary patina. Preserving this patina especially the cuprite, along with surface details, is the main goal in the conservation of copper-based coins^6,15^.

Patina is a thin, natural or artificial layer that forms on bronze due to chemical reactions such as oxidation^16^. It develops over time through exposure to environmental factors, including humidity, pollution, and soil. Patinas are composed of compounds such as copper oxides (e.g., cuprite), carbonates (e.g., malachite), or sulfates^17,18^. Natural patinas provide protection against corrosion, while artificial patinas are applied for aesthetic or protective purpose. In archaeology, patinas are valuable as they act as a historical record and serve as a protective barrier, making their preservation crucial in conservation efforts. Surface degradation occurs due to corrosion, which involves not only oxidation but also the formation of copper chlorides (bronze disease) and other corrosion products that vary in thickness and composition^19^.

Bronze disease is an active corrosion process in which bronze reacts with chloride ions to form cuprous chloride (CuCl), leading to metal degradation. In humid environments, cuprous chloride reacts with water to produce hydrochloric acid, further accelerating deterioration. This results in pitting and flaking, weakening the structural integrity of artifacts.^20–22^, Bronze disease can compromise the structural, historical, and aesthetic values of archaeological coins. The reaction speed on the bronze surface varies depending on the object’s history and surrounding environment^4,23^. Sulfur and chloride ions can penetrate the patina and metal core, causing pits to form on the surface. Aggressive chloride accelerates the dissolution of copper-based alloys, causing the loss of native protective layers in burial environments^24^. The corrosion layers mainly consist of malachite (CuCO_3_. Cu(OH)_2_), which disrupts the CuCl/Cu_2_O mixture and causes exfoliation. Post-excavation cuprous chloride (CuCl) hydrolyzes into hydroxyl chloride polymorphs Cu_2_OH_3_Cl in the presence of water^25^. Bronze disease occurs with the formation polymorphous copper hydroxyl chlorides, including atacamite, paratacamite, clinoatacamite, and botallackite^26,27^.

Lead plays an effective role in influencing the corrosion rate by forming highly stable lead carbonates and insoluble lead chloride, which remain stable after excavation^28^. Protective conservation is the most effective process to prevent or at least delay degradation. However, achieving suitable environmental conditions is challenging, making it necessary to apply a protective system^29^. Protective coatings, including natural patinas or applied treatments, inhibit corrosion by forming a barrier against moisture, oxygen, and pollutants, helping to preserve the artifact and minimize further damage. The coating system is a significant topic to stop corrosion and ensure the long-term stability of archaeological coins. There is a continuous study to provide better protection for objects by preventing the influence of humidity and contaminations^30,31^ with special requirements for conservation-restoration ethics such as ease of application, transparency, good appearance, long-term stability, reversibility, and safety for conservators-restorers and the environment^32^.

Through ancient conservation, natural resins were used to coat metal surfaces, but they often curled and flaked away. In contrast, methyl acrylate/ ethyl methacrylate 30:70% (Paraloid B-72®) is considered the most widely used coating for archaeological objects in Egypt due to its economic efficiency, transparency, and reversibility^33–35^. It can be applied on both clean metal surfaces and those covered with patina^36,37^.

The study of archeological coins is essential for both the preservation of cultural heritage and advancements in corrosion science^38^. Analyzing the corrosion products of bronze archeological coins provides insights into the surface morphology, which aids in the preservation of cultural heritage.

The elemental composition of these corrosion products was analyzed using established methods, including scanning electron microscopy (SEM) with energy dispersive X-ray spectroscopy (EDX). X-ray diffraction (XRD) was employed to identify specific corrosion products on the surface. Stereo microscopy was used to identify the heritage of the coins and to visualize the corrosion and mineral layers on their surfaces. This study also aims to assess the effectiveness of bronze patina and/or coatings, both fresh and aged (the short-term effectiveness of Paraloid as a protective film), in aggressive environments using electrochemical techniques such as potentiodynamic polarization (PDP), electrochemical impedance spectroscopy (EIS), and cyclic voltammetry (CV).

## Experimental

### Description of archaeological coins

A collection of 300- Greco-Roman bronze coins and small fragments was excavated from a pottery pot buried in humid, salty soil at Karnak Temple. The coins suffered damage in the burial environment and were covered with thick layers of corrosion products adhered to soil encrustations. Some coins became worn and fractured due to reactions with oxygen and chloride ions in the humid environment.

This study focuses on bronze fragments found in similar condition as the coins. These fragments are small, lack detailed features, and contain naturally formed corrosion products mixed with soil deposits.

### Appropriate protocol of coin treatment

#### Cleaning

Cleaning is often the initial step in many conservation processes and is regarded as one of the most challenging tasks in preserving metal artifacts. It requires great care to preserve the coin’s original form, function, and material. For this reason, efforts should be made to develop improved techniques and methods specifically designed for cleaning corroded archaeological coins^39^.

### Mechanical cleaning

The commonly adopted procedure primarily involves mechanical cleaning^40,41^, which must be performed with great care to avoid surface damage that could lead to the loss of material or important historical information**.** Mechanical cleaning of coins is a delicate process that requires careful consideration of the material, historical significance, and the type of corrosion present. Appropriate tools must be selected to ensure minimal damage. These cleaning treatments involve the physical removal of corrosion products and encrustations to reveal the original surface of the coin. They are typically combined with other conservation methods to preserve the coin’s integrity and historical value. Precision tools like scalpels, fiberglass brushes, dental picks, and needles are employed to scrape away corrosion, soil deposits, and dirt. Soft brushes and micro-scarpers are used gently to avoid scratching the surface while removing foreign materials from details and decorated areas.

### Chemical cleaning

Dilute hydrochloric acid was used to remove thick soil and corrosion encrustations from the surface. EDTA disodium and glycerol alkaline solution were also employed in the chemical cleaning process to eliminate corrosion products and chloride compounds^42^ such as those associated with bronze disease which were mixed with earthen encrustations^19,40,43^.The fragments were cleaned using a protocol similar to that used for coin cleaning focusing on the aesthetic form^6,44^, as shown in Table 1. For better treatment, distilled water was used to eliminate any residual effects of the chemical solutions, followed by alcohol to remove any remaining residues.

**Table 1:** Stepwise procedure of the chemical treatment of the archeological coins.

Chemical solution	Conc	The goal of treatment	Duration	Temperature
Hydrochloric acid	3%	Soil encrustations	30 min	Room temperature
Hexameta phosphate (50 ml distilled water + 75 g Hexameta phosphate )	2%	Clay encrustations	30 min	Room temperature
Citric acid	2%	Soil encrustations	30 min	60 °C
EDTA tetra sodium	3%	Green patina + additional treatment	1 h	Room temperature
EDTA disodium	3%	Bronze disease	1 h	Room temperature
Glycerol Alkaline (10 ml glycerin + 100 ml distilled water + 3 g sodium hydroxide)		Bronze disease + additional treatment	3 h	Room temperature

#### Coating

Once the surface was cleaned, the coating process began by applying two layers of 3% Paraloid B-72®. Acetone was selected as the solvent for the solid resin due to its lower hazard compared to other solvents^33^. A smooth and uniform surface was achieved by brushing in one direction, followed by brushing in the opposite direction. To enhance adhesion, coatings were applied as multilayer films. The first layer ensured good adhesion to the metal surface, the second layer provided protection, and the outer layer faced environmental risks^10^. Once dried, Paraloid B-72® formed a clear, transparent film.

### Sampling

The visual observation of surfaces covered with similar corrosion products prompted the selection of samples for SEM and EDX investigation to provide insight into the morphology and an estimation of the expected patina and bronze disease. Experiments were conducted on a set of archaeological coin fragments selected from a group of excavated objects. The coin fragments for each sample were divided as follows:

1. Bronze disease fragments labeled as (BD) contain high a content of chloride ions.
2. Patinated fragments covered naturally with Cuprite labeled as (P).
3. Patinated, treated, and past-coated fragments, which were brushed with 3% Paraloid B-72® and preserved inside polyethylene pages for two years, labeled as (S1 and S2),
4. Patinated, treated, and freshly coated fragments, which were freshly brushed with 3% Paraloid B-72® labeled as (S3).

### Surface characterization

Chemical characterization is considered a vital source for advancing the understanding and inhibition of corrosion. Multi-investigation and analytical techniques provide valuable information relevant to archaeological coins and conservation treatment. The visual appearance of the fragments extracted from the burial environment was examined using the Leica S9i, Stereo Microscopy(SM).

The surface morphology was investigated using non-destructive techniques with high resolution scanning electron microscopy (**SEM**) equipped, coupled with Energy Dispersive X-ray spectroscopy (**EDX**) (Quanta FEG 250 with field emission gun FEI, Netherlands). The operating parameters were as follows: accelerating voltage, 20 kV; accumulation time, 100 s; and window width, 8 μm at Central Service Labs (NRC). The samples were coated with gold to overcome the effect of sample charging in the electron beam using an S150A Sputter Coater System from Edwards.

Powdery corrosion products obtained during the mechanical cleaning of the coin surfaces were analyzed using the X-ray diffraction (XRD). The XRD equipment (PAN Alytical, Netherlands) at the Central Metallurgical Research and Development Institute (CMRDI) operated over a 2θ range of 15–80° with a scanning rate of 2°/min. This method is highly effective for identifying the chemical composition and crystal structure, which helps characterize the bronze patina and corrosion compounds.

### Electrochemical methods

Electrochemical techniques such as electrochemical impedance spectroscopy (**EIS**), potentiodynamic polarization (**PDP**), and cyclic voltammetry (**CV**) were employed to study the behavior of bronze patina and/or protective films.

These techniques were carried out using Autolab potentiostat /galvanostat PGSTAT 302N connected to a computer, in a classic three-electrode design cell. The counter electrode was platinum, and the reference electrode was silver/silver chloride. Different fragments under study (BD, P, S1, S2, and S3) served as the working electrode and were exposed to an electrolyte solution of 0.6 M NaCl at room temperature. The surface area of each sample was measured precisely before testing. Although the areas were nearly equal, any small differences were accounted for by dividing the total current by the exact surface area to normalize the current, obtaining current density (A.cm^−2^).

#### Electrochemical impedance spectroscopy (EIS)

EIS is a non-destructive tool used in conservation and restoration treatments for evaluating the protective coatings of metallic cultural heritage^45^. EIS was carried out in the frequency range of 100 kHz to 0.01 Hz using an AC source with an amplitude of 10 mV peak to peak at open circuit potential (OCP). The Nova 1.11 software was used to fit the experimental data to an appropriate electrical circuit***.***

#### Potentiodynamic polarization (PDP)

All samples were immersed in 3.5 wt. % NaCl solutions for 1 h until constant open circuit potential (OCP) values were achieved^9^. Potentiodynamic polarizations were conducted by varying the potential ± 250 mV from the OCP with a scan rate of 1mVs^−1^.The Nova 1.11 software was used to estimate the electrochemical parameters, including the corrosion current density (*I*_corr_), corrosion potential (*E*_corr_), polarization resistance (R_pol_), and Tafel slopes (*β*_a_, *β*_c_). To assess the protective effectiveness of the coating, the following formula was used for the calculation:

1 $$PE\% = \frac{{I_{0} - I_{C} }}{{I_{0} }} \times 100$$

where, *I*_o_ and I_c_ are the values of the corrosion current densities of uncoated and coated samples.

#### Cyclic voltammetry

Cyclic voltammetric analysis was performed from negative to positive directions over a potential range from − 0.2 to 1.0 V, with a scan rate of 1 mVs^−1^, to evaluate the behavior of the coating and the corrosion products on the surface.

## Results and discussion

Archaeological coins play a crucial role in providing archaeologists with valuable insights, often surpassing those offered by written documents, due to their symbols and concise inscriptions^46^. Understanding the chemical composition, patina characteristics, and corrosion attributes of these coin’s surfaces is vital. Our investigation of coin fragments is significant and presents a challenging task in both corrosion science and cultural heritage preservation.

Numerous studies have highlighted the importance of analyzing archaeological coins. For instance, research by Ling et al. investigated the archaeological round bronze coins to disclose their chemical composition, nature of the patina and corrosion features on the coin surface using XRD and SEM equipped with EDX^13^ .

Employing non-destructive and micro-destructive techniques such as XRD, SEM– EDX, and Stereo Microscopy were strategic in minimizing mechanical interventions on the archaeological artifacts. The choice of the methodologies was effective for characterizing the corrosion products, as the techniques provided complementary information^47^. These studies highlight the necessity of detailed chemical and morphological analyses to protect and preserve our cultural heritage.

### Coin treatments

The results of visual observation revealed that the coins, prior to cleaning, were severely deteriorated, with an extensive corrosion layer obstructing their surface. The surfaces exhibited a rough, corroded texture, characterized by cracks. Additionally, various types of corrosion products in different colors were observed, and the surfaces were covered with soil residues.

**Fig. 1:**
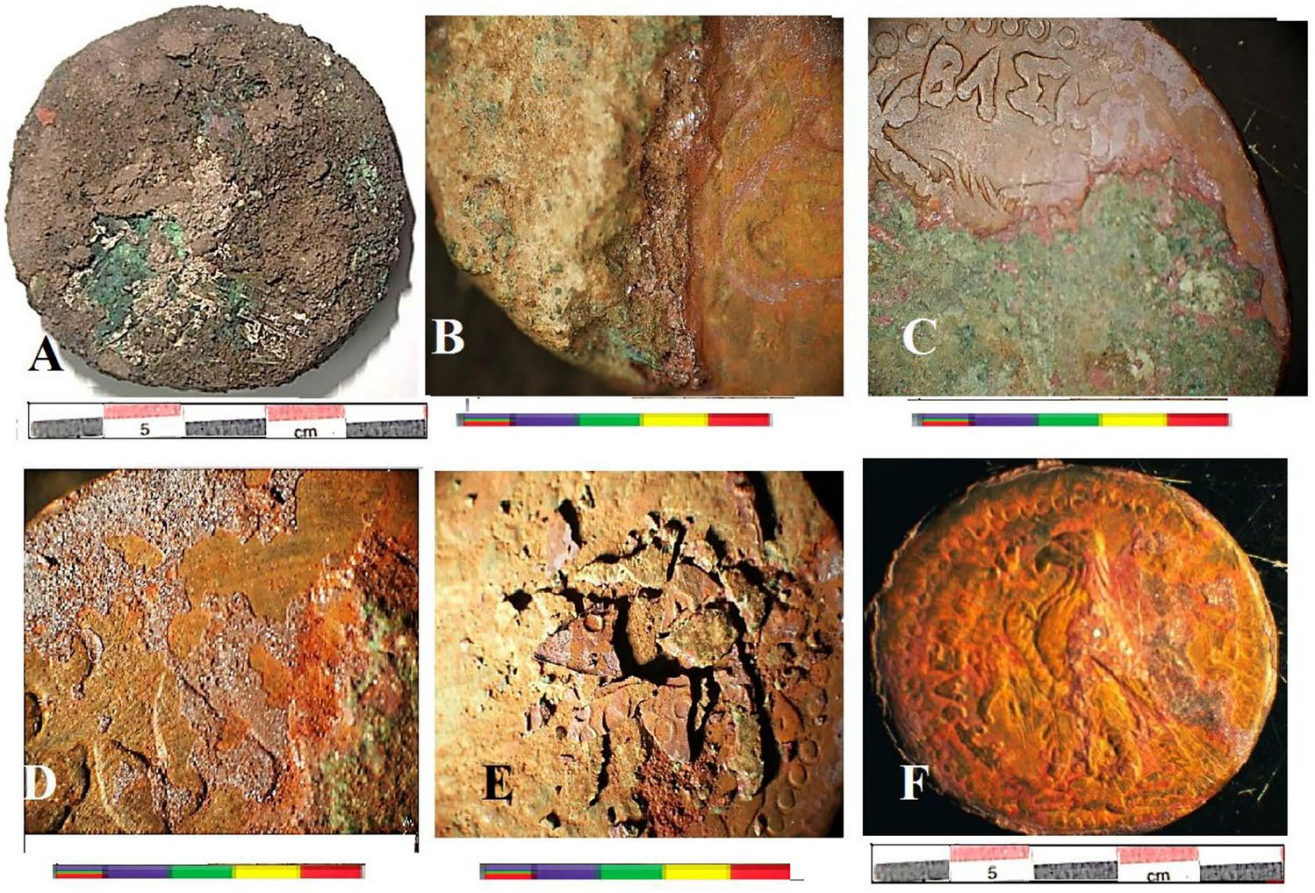
The archaeological excavated coin: (**A**) before treatment, (**B**, **C**) coin edges (**D**, **E**) after mechanical cleaning, and (**F**) after complete treatment.

The coins were encrusted with thick layers of corrosion products tightly adhered to soil deposits. These corrosion products, predominantly brown copper oxides, completely covered the surface interface, as shown in Fig. 1^19^. Cleaning is the crucial initial step in many conservation processes. It poses a significant challenge in preserving metal coins. It involves the careful removal of corrosion products and soil sediments to reveal the details of the coins, as described in the experimental section.

Figure 1A shows the coin’s appearance before treatment, covered with heterogeneous, thick layers of crust in varying colors, where corrosion products coexist with soil particle. Figures 1B and C illustrate an edge of coin revealing layered corrosion. Figure 1D and E depict the coin after mechanical treatment, with the external patina removed, as shown in Fig. 1E. After cleaning, the coin was left with a thin layer of corrosion products, which revealed the surface morphology more clearly, along with a homogeneous cuprite patina after complete treatment, as shown in Fig. 1F.

Upon completing the conservation treatments, the surface exhibited roughness. Therefore, polishing was necessary to enhance surface details, resulting in an improved appearance.

### Stereo microscopy(SM)

Stereo microscopy plays a crucial role in identifying the heritage of the coins and visualizing the corrosion and mineral layers present on their surfaces. The visual appearance of the coins is highly significant, particularly in conservation and restoration efforts within cultural heritage. Therefore, it is essential that the coatings applied to metals do not alter their visual aspects. A diagnostic investigation of the surface appearance of the fragments was conducted using stereo microscopy. Observations using the SM revealed the morphological characterization of the fragments, highlighting the color features of corrosion products overlapping earthen sediments. Coins exposed to saline soil developed a thick layer of corrosion. This layer, referred to as malignant patina, typically exhibits brownish-green or greenish-blue, as observed through SM^46^.

**Fig. 2:**
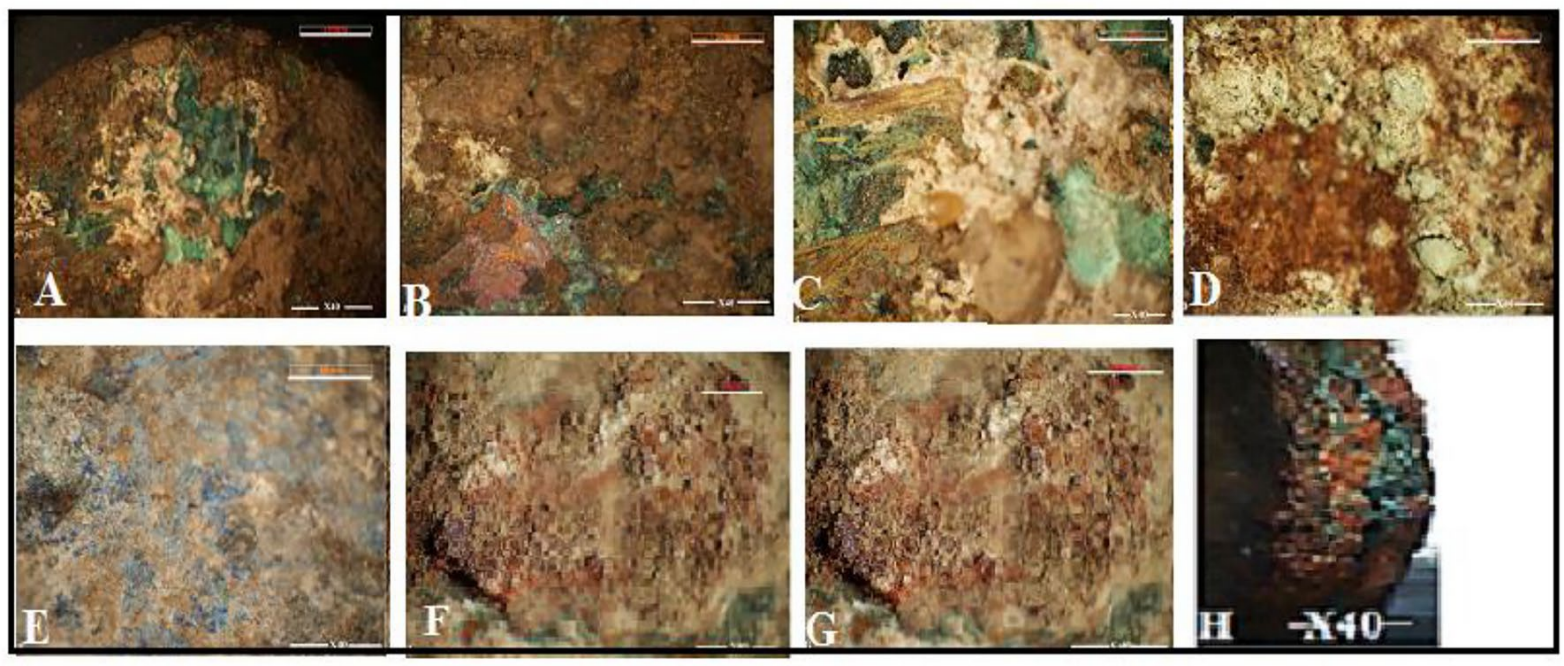
Stereo microscopy images of the fragments show (**a**) at X20 magnification, the morphological characterization based on color features of corrosion products overlapping earthen sediments, (**b**–**d**) at X40 magnification, (**C**, **D**) organic soil adhered to corrosion products, (**E**) Blue-colored azurite adhering to soil deposits, (**F**, **G**) soil encrustation and, (**H**) edge of coin of the round coin after treatment.

Changes in the morphology, thickness, and surface color of the excavated coins were attributed to corrosion products on their surface. Vitreous deposits and patches appeared green with dark brown areas as shown in Fig. 2A–D. Corrosion products, combined with burial deposits, were observed covering most of coins’ surfaces. Investigations confirmed that the thick encrustation primarily consisted of a mixture of soil and corrosion deposits.

Figure 2A and B show the earthen sediments covering the green patina. Figure 2C and D display organic soil adhered with corrosion products. In Fig. 2E blue-colored cupric carbonates (azurite) are visible, adhering to the soil deposits. Figure 2F and G. depict soil encrustation, while Fig. 2H illustrates an edge of round coin after treatment.

These components contribute to soil layers that can induce stress corrosion cracking under the combined influence of tensile mechanical stresses and a corrosive environment. Cracks may appear as individual or branched, inter-granular or trans-granular^48^.

During the corrosion process, volume changes occur, resulting in cracks along the patina, which facilitate the diffusion of soil elements into the internal areas of the metal.

Additionally, other components in the bronze alloy, such as lead, significantly impact the corrosion rate. Lead forms highly stable lead carbonates and insoluble lead chloride, which remain stable, even after excavation.

### Scanning electron microscopy and energy dispersive X-ray spectroscopy (SEM–EDX)

**Fig. 3:**
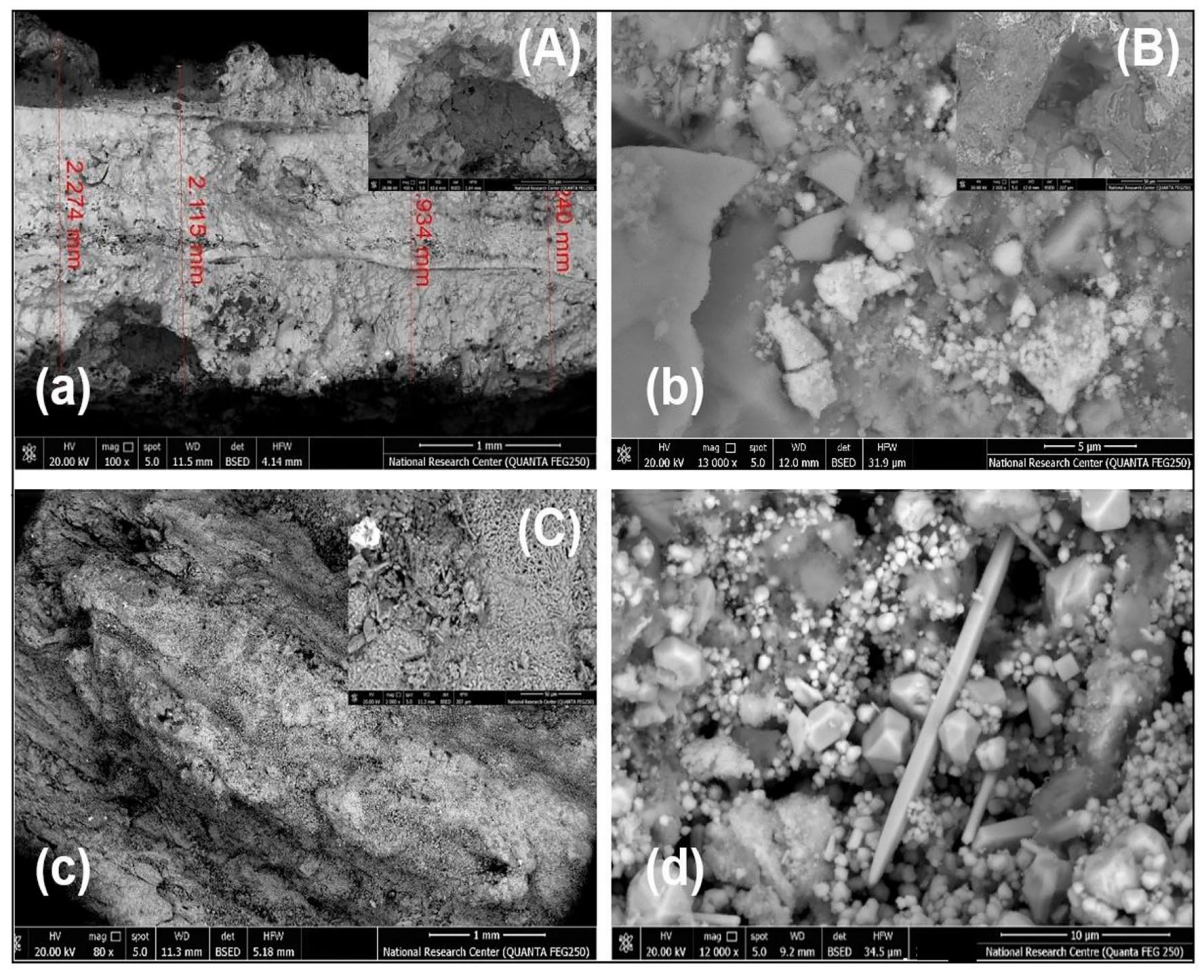
SEM images of different coins with low and high magnfications (**a**) BD, (**b**) S1, (**c**) S2, (**d**) corrosion product adehered to soil.

SEM–EDX was utilized to study surface morphology, providing insights into the corrosion processes and characteristics of buried metals. Coin fragments were investigated using non-destructive techniques like SEM. The results confirmed that the pits were filled with chloride-corrosion products mixed with earthen materials, as shown in Fig. 3. SEM images (a and A) display a cross-section of bronze disease (BD) at different magnifications (low and high). SEM images (b and B) for the past coated sample S1 reveal fragile minerals on the surface, also at varying magnification (low and high). SEM images (c and C) of the S2 sample show pitting corrosion and dendrites on the coin surface, again at both low and high magnifications. Magnification of SEM image (d) illustrates crystals of corrosion products adhered to soil sediments.

Upon inspection of these figures, it becomes evident that chloride ions have caused a complete transformation of the metal into corrosion products, with soil elements adhering to the surface^49^.

**Fig. 4:**
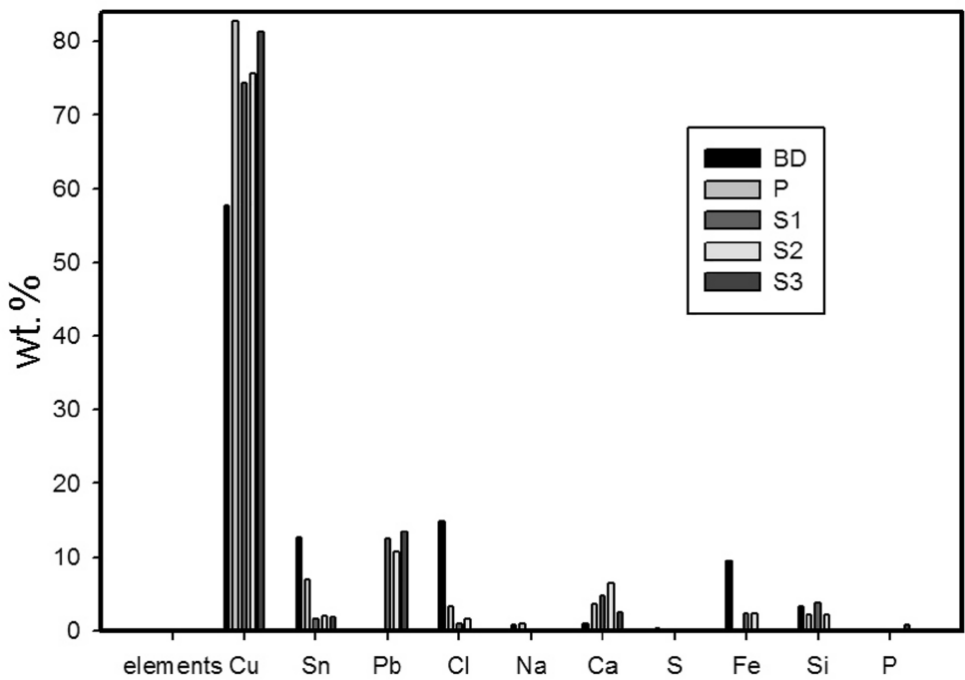
Chart of the concentration (wt. %) of the detected elements in all samples.

The EDX investigation conducted on the fragments, as listed in Table 2 and represented in supplementary Fig. S1, demonstrates that the coin fragments are composed of ternary alloys consisting of copper, tin, and lead. Figure 4 represents the weight percentages of various elements across all samples. Higher concentrations of iron in the crusts were attributed to contamination from soil oxides. Silicon, identified as the major foreign element on the surface, referring to the burial environment. Sulfur, typically present in lower percentages compared to atmospheric conditions, was detected in all fragments^50^.

**Table 2:** EDX quantitative results for the different surface regions of the fragments.

Sample	Elements (wt. %)									
	Cu	Sn	Pb	Cl	Na	Ca	S	Fe	Si	P
BD	67.13	12.84	0.02	15.03	0.79	0.86	0.16	1.07	2.09	0.02
	57.79	12.58	0.02	14.87	0.70	0.90	0.30	9.49	3.32	0.03
P	82.68	6.96	0.01	3.32	0.88	3.67	0.24	0	2.24	0
S1	74.35	1.57	12.40	0.89	0	4.67	0.01	2.28	3.83	0
S2	75.52	2.07	10.81	1.54	0	6.42	0	2.41	2.23	0
S3	81.29	1.84	13.46	0	0	2.51	0	0.17	0	0.73

The high concentration of tin observed at the surface of corroded bronzes (BD) is attributed to copper depletion and the subsequent formation of cassiterite (SnO₂). This concentration gradually decreases from the outer layers towards the alloy core. The exterior layer primarily consists of Cu (II) compounds and soil material, followed by a fragmented layer of cuprous oxide. The surface layer of the alloy exhibits copper depletion, tin enrichment, and elevated chloride ions levels^51–54^.

The BD sample exhibited low concentrations of lead and relatively high level of tin and chloride. In contrast, Past coated samples S1 and S2 displayed lower chloride concetrations. Additionally, the detection of light elements, such as oxygen, may have been underestimated compared to heavier elements^55^.

After cleaning and desalination, the treated coin fragments showed reduced chloride content compared to untreated fragments. In the freshly coated sample, chloride ions were completely removed. This suggests that incomplete treatment was the primary cause for the presence of chloride ions. Empirical evidence indicates that corrosion can persist even after protection, particularly in environments with relative humidity exceeding 40%.

### X-ray diffraction (XRD)

XRD analysis provided valuable insight into the chemical structure of the metal objects and soil encrustation. Table 3 summarizes the results, showing the d-spacing values and best matching reference cards obtained from the analysis.

**Table 3:** X–ray diffraction results.

XRD results of bronze fragments covered with green patina			Identification of corrosion products	
Pos.[°2θ.]	d-spacing A^o^	Crystalline components	Phase	International powder diffraction files JCPDS
20.98	4.23	Chalcoalumite	CuAl_4_SO_4_(OH_12_).H_2_O	8–0142
26.78	3.33	Quartz	SiO_2_	46–1045
27.87	3.20	Orthoclase	KAlSi_3_O_8_	09–0462
42.52	2.13	Atacamite	Cu_2_(OH)_3_Cl	2–0146
44.46	2.04	Malachite	Cu_2_(OH)_2_CO_3_	04–0836
60.07	1.54	Bronze	(Cu, Sn)	2–0004
XRD results of bronze disease			Identification of corrosion products	
Pos.[°2θ]	d-spacing A^o^	Crystalline components	Phase	International Powder diffraction files JCPDS
16.15	5.48	Paratacamite	Cu_2_(OH)_3_Cl	15–0694
32.32	2.77	Paratacamite	Cu_2_(OH)_3_Cl	2–0146
36.39	2.47	Cuprite	Cu_2_O	05–0667
39.67	2.27	Clinoatacamite	Cu_2_(OH)_3_Cl	15–0490
42.27	2.14	Paratacamite	Cu_2_(OH)_3_Cl	15–0694
50.10	1.82	Atacamite	Cu_2_(OH)_3_Cl	80–0392

The XRD data indicates that the BD fragments primarily consists of hydroxyl chlorides, such as paratacamite Cu_2_(OH)_3_Cl, chalcoalumite CuAl_4_SO_4_ (OH_12_).H_2_O, with atacamite being the dominant corrosion products on the surface. The interior surface contains corrosion products beneath external soil sediments, including quartz and orthoclase. Malachite, a basic copper carbonate, was also identified on the outermost layer of the fragments.

Table 4 compares the XRD results for all fragments. The main corrosion products identified such as cuprite, atacamite, paratacamite, and chalcoalumite, align with those commonly reported in the literature as primary constituents formed in the burial environment. Additionally, quartz and orthoclase were detected^3^. In the freshly coated fragments, corrosion products were significantly reduced.

**Table 4:** XRD results of the different studied fragments.

Crystalline components	Phase	International powder diffraction files JCPDS	Samples				
			BD	P	S1	S2	S3
Cuprite	Cu_2_O	04–0836	**++**	** + + + **	** + + **	** + + **	** + + + **
Atacamite	Cu_2_(OH)_3_Cl	2–0146	** + + + + **	** + **	** + **	** + + **	**–**
Paratacamite	Cu_2_(OH)_3_Cl	2–0146	** + + + + **	** + + **	** + **	** + **	**–**
Chalcoalumite	CuAl_4_SO_4_(OH_12_).H_2_O	8–0142	** + + + **	**–**	**–**	**–**	**–**
Quartz	SiO_2_	46–1045	** + + + + **	** + + **	**–**	**–**	** + **
Orthoclase	KAlSi_3_O_8_	09–0462	** + + + + **	**–**	**–**	**–**	**–**

+  +  +  + very high amount**, +  +  + high, +  + **Moderate amount,** + **Trace amount

The typical corrosion processes affecting bronze artifacts involve the formation of various oxides and carbonates. The primary reactions include the formation of cuprite (Cu_2_O), tenorite (CuO), malachite (Cu_2_(CO_3_)(OH)_2_), and cassiterite (SnO_2_)^56^. These reactions occur under specific environmental conditions and significantly influence the preservation of bronze artifacts. For instance, cuprite acts as a protective layer inhibiting further corrosion, whereas cassiterite forms through the oxidation of tin and resulting in a brittle protective layer. Understanding these processes is crucial for developing effective conservation strategies^4^.

The initial stage of corrosion in copper and its alloys involves the formation of a passive oxide layer, which may occur even before burial. Once buried, copper typically undergoes preferential corrosion due to the aggressive conditions in the soil, with copper being redeposited onto the surface. The first oxide layer that forms at the copper-soil interface is typically cuprite (Cu₂O), also known as the Primary Patina^57^

2 $$4{\text{Cu }} + {\text{O}}_{2} \to 2{\text{Cu}}_{{2}} {\text{O}}$$

Under burial conditions, copper is more likely to undergo reactions involving CuCl in an aerobic and moist environment. When copper comes into contact with water in these conditions, it results in the formation of basic copper chlorides, such as atacamite and paratacamite. These compounds are common corrosion products that develop as part of the ongoing degradation process in such environments^13^.

Bronze Disease (Atacamite Formation)

3 $$2{\text{Cu}}_{2} {\text{O}} + {\text{O}}_{2} + 2{\text{Cl}}^{ - } + \, 4{\text{H}}_{2} {\text{O}} \to 2{\text{ Cu}}_{2} {\text{Cl }}\left( {{\text{OH}}} \right)_{3} + 2{\text{OH}}^{ - }$$

### Electrochemical measurements

Potentiodynamic polarization (PDP), electrochemical impedance spectroscopy(EIS), and cyclic voltammetry (CV) are electrochemical techniques offering a quantitative analysis of the corroding metals^58^. These methods were employed to evaluate the corrosion resistance of different samples, including bronze disease (BD), patinated (P), sample coated two years ago (S1, S2) and freshly coated patinated sample (S3), under aggressive environmental conditions.

#### Electrochemical impedance spectroscopy (EIS)

**Fig. 5:**
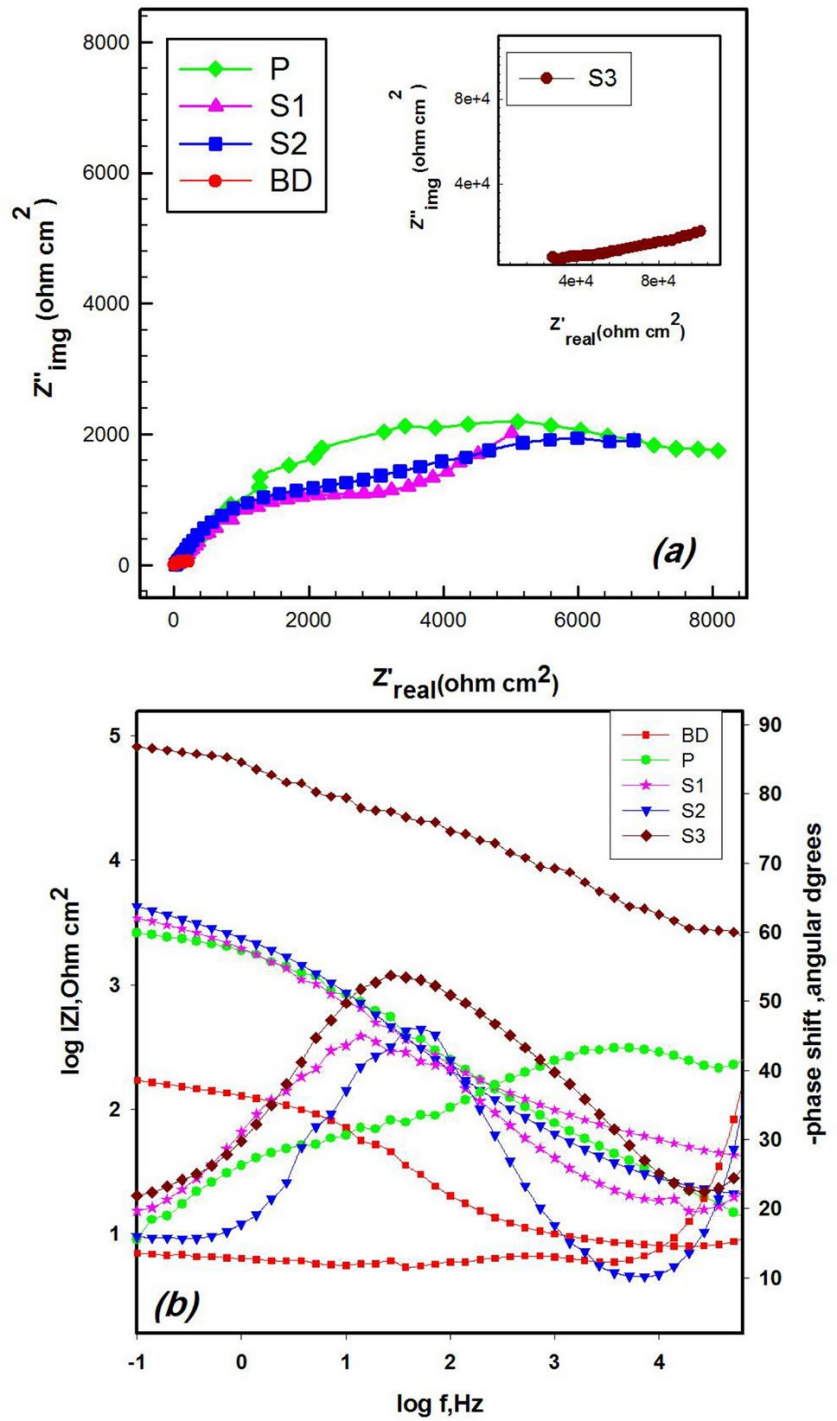
**(a)** Nyquist plots and (b) Bode and Phase plots of BD, P, S1, S2 and S3 samples of the studied coin fragments in NaCl solution.

Electrochemical impedance spectroscopy (EIS), a non-destructive technique, was employed to study the electrode/electrolyte interface and the corrosion processes occurring on the fragment surfaces^58^. EIS measurements were recorded at the open circuit potential (OCP). Figure 5a shows the Nyquist spectra for all samples. The Nyquist plot of the BD sample exhibits an arc shape, indicating the diffusion of oxygen and electrolyte through pores on the surface.

The patinated sample (P) displays a semicircle in the Nyquist plot, indicating that the patina, consisting of a native oxide film protects the coin by preventing the penetration of chloride ions. This implies that the corrosion process does not occur under diffusion control with corrosion products presumably Cu_2_O, accumulating at the interface. The past coated samples (S1 and S2) and freshly coated sample (S3) also show arc shapes, with the S3 fragment demonstrating the largest diameter. This indicates that the fresh coating effectively prevents the penetration of corrosive species, leading to higher resistance^59,60^.

The Bode plot Fig. 5b shows the relation of log |*Z*|/Ω *cm*^2^ with log f/Hz. At high frequencies, the solution resistance is dominates, while at low frequency, coating resistance is dominant. The middle frequency range represents the capacitive region. Over the frequency range used, S3 displays a higher impedance modulus, |*Z*|, indicating a stable, compact external layer that effectively protects the bronze from further corrosion^61,62^.

In the case of a damaged film (BD), the impedance modulus, |*Z*|, exhibits low values, and a plateau emerge at medium frequencies. This suggests that the corrosion layer is porous and ineffective in protecting the underneath substrate. The surface has not yet reached a stable condition, indicating that the corrosion process is still in progress. The phase angle changes from 40 to 0°, involving mass transfer diffusion effects. The higher impedance of the patinated sample (P) compared to BD indicates that the surface is covered by a more homogeneous and well-adherent native oxide layer, providing better protection than BD^63^.

For the S1 and S2 past coated coin fragments, the impedance modulus is slightly higher than that of the patinated sample (P), and lowers than that of S3. This is due to the presence of both oxide and the coating film; however the coin surfaces were affected and partially damaged by the aggressive environment.

The phase angle (θ), plotted against log frequency, is shown in Fig. 5b. The phase angle indicates the difference in phase between the applied AC voltage and the resulting current. It provides insight into the capacitive and resistive behavior of the electrochemical system. A high phase angle (close to 90 degrees) suggests that the system behaves like a capacitor, indicating the presence of a dielectric layer, such as a passive oxide layer or coating. Conversely, a low phase angle (close to 0 degrees) suggests that the system behaves like a resistor, indicating the presence of resistive elements like corrosion products.

As shown in the Figure, broad phase distributions are represented by two-time constants. The phase angle shifts to higher frequencies, with maximum value observed in S3 indicating a high level of corrosion protection.

**Fig. 6:**
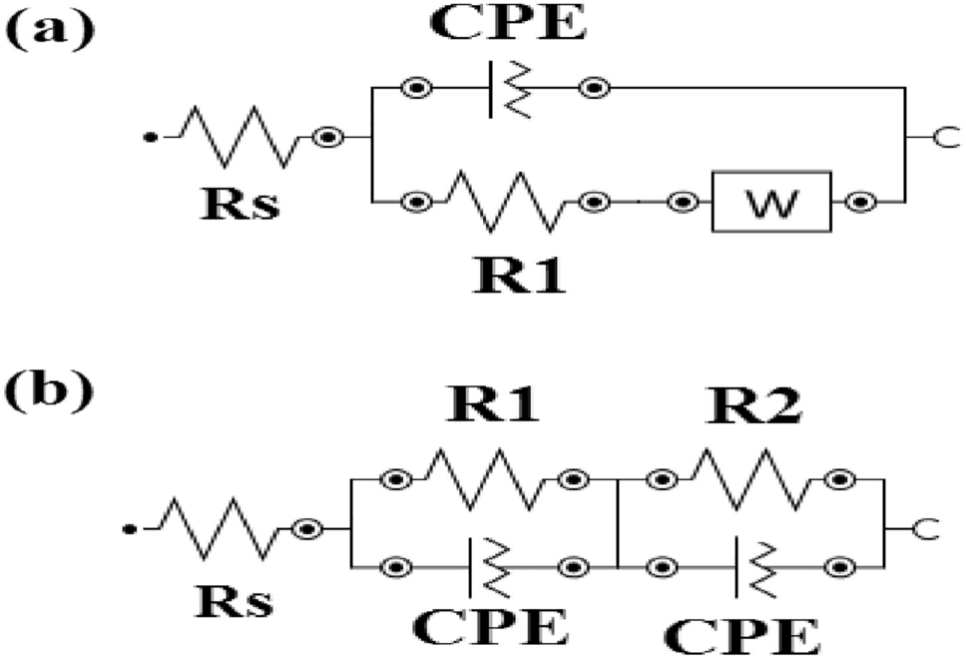
The fitting circuits (**a**, **b**) of different coin fragments in NaCl solution.

The impedance spectra were analyzed by fitting the data to the equivalent electrical circuit as shown in Fig. 6a, b.The fitting quality of the suggested circuit has been verified by the chi-square (*χ*2) values listed in Table 5. A smaller χ2 value indicates a better fit of the recommended equivalent circuit.

**Table 5:** The parameters obtained from EIS for all samples in 0.6 M NaCl at 25 °C.

Sample	*R*_s_ Ω	*R*_1_ Ωcm^2^	CPE_1_ Yo (Ω^−1^ cm^−2^S^n^)	n	*R*_2_ K. Ω cm2	CPE_2_ Yo (Ω^−1^ cm^−2^S^n^)	n	W Ohm cm^2^	χ^2^
BD	7.8	140	6.7 × 10^–4^	0.55	–	–	–	0.036	0.15
P	28.7	914	3.8 × 10^–5^	0.72	6.5	1.45 × 10^–5^	0.65	–	0.19
S1	13.2	1379	5.42 × 10^–5^	0.88	10.1	3.17 × 10^–4^	0.68	–	0.02
S2	21.9	1333	4.97 × 10^–6^	0.81	70.7	1.02 × 10^–5^	0.70	–	0.022
S3	131	2300	5.25 × 10^–8^	0.89	400	1.07 × 10^–5^	0.75	–	0.012

The EIS parameters are listed in Table 5^64^. The resistance of the electrolyte (*R*_s_) is in series with a constant phase element (CPE1), which is in parallel with another resistor (R1), representing the properties of the coating. Additionally, this in series with a circuit constituted by CPE2 and R2 elements in parallel modeling the double-layer capacitance and the charge transfer resistance, respectively^45^, as well as the Warburg impedance (W), which models a diffusion process^65,66^. A constant phase element is used instead of a capacitor due to surface roughness and irregular distribution^45,67^. The impedance of a CPE is defined by the empirical expression:

4 $$Z_{{{\text{CPE}}}} = \left( {\frac{1}{{Y_{o} \left( {j\omega } \right)^{n} }}} \right)$$

Where, *Y*_*o*_ is the magnitude of CPE, j = (−1)^1/2^, ω = 2πf and n is the exponent. The value is − 1 ≤ α ≤  + 1. When n = 0, the CPE is a resistor; when n = 1, the CPE is a capacitor and when n =  − 1, the CPE is an inductor. Finally, if n = 0.5, the CPE is the Warburg impedance, i.e. it models a diffusion process^66^.

In general, higher resistance values and lower CPE element values, coupled with phase angle parameters, suggest effective coating protection. The CPE parameters accommodate deviations from ideal capacitive behavior, providing a more comprehensive insight into coating’s protective efficiency^2^.

From Table 5 the highest resistance value was recorded for S3. Skale et al. have proposed that the diffusion of species through the pores of the surface^68^ can be modeled using the Warburg impedance (*W*), which was recorded for BD^69^. The exponent (n) of CPE is around 0.55 and the low R_ct_ values are expected in the presence of BD due to the corrosion process is still in progress, involving mass transfer diffusion effects (diffusion of oxygen and/or electrolyte through pores or defects in the surface indicating the inhomogeneity surface^45^. On the other hand, the values of the exponent (n) of CPE range from 0.77 to 0.89, indicating the reduction of surface roughening attributed to the behavior of the coating.

The uncoated surfaces facilitate greater electrolytes penetration, thereby increasing the contact area between the electrolyte and the underlying electrode. This can potentially reduce solution resistance due to enhanced ionic conductivity within the pores. In contrast, coated surfaces restrict ion dissolution, resulting in higher solution resistance^45^.

#### Potentiodynamic polarization

**Fig. 7:**
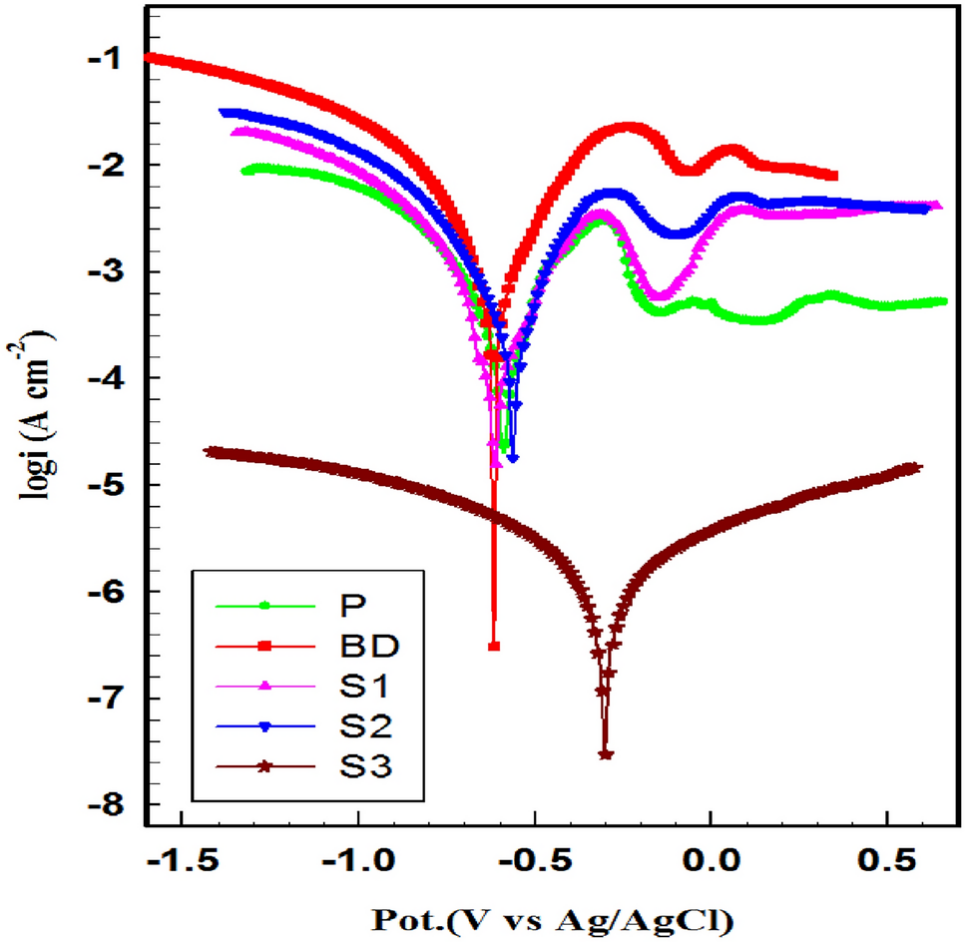
Potentiodynamic curves for different coin fragments in 0.6 M NaCl.

Figure 7 illustrates the PDP curves of coin fragments studied after 1 h of immersion in 0.6 M NaCl solution^70^. Tafel plots offer graphical insight into the electrochemical behavior of materials, aiding in the understanding of their corrosion characteristics. By analyzing the Tafel curves (The logarithmic current density against the electrode potential) of various samples, including BD, P, S1, S2, and S3 depicted in Fig. 7, it is evident from the Tafel plots that both anodic and cathodic curves shift toward lower current densities for the patinated (P) and the coated fragments (S1, S2, and S3) compared to the uncoated fragment (BD)^71^.

The decrease in anodic current density is more pronounced than that of the cathodic current density. Additionally, the corrosion potentials of the patinated and coated fragments show a significant shift towards more noble values compared to the BD fragment. This suggests that the coating provides a protective barrier, preventing electrolytes from penetrating the surface, thereby reducing active area and delaying the corrosion reactions (metal dissolution and oxygen reduction), which ultimately slows down the corrosion rate (CR)^28^.

Upon examining the Tafel curves, it is evident that Sample S3 exhibits distinct behavior compared to the others. The Tafel curve for S3 shows a significantly different slope and shape indicating variations in the electrochemical reactions occurring on its surface. Notably, the shift in corrosion potential (E_corr_ vs. Ag/AgCl), from -0.61 of BD to -0.314 V for S3 suggests a more noble (less reactive) behavior. Additionally, the anodic and cathodic branches of S3 Tafel curve differ significantly from those of other samples, indicating differences in the oxidation and reduction reactions taking place on its surface.

Electrochemical parameters calculated by Nova 1.11 are listed in Table 6, including corrosion potential (*E*_corr_), the corrosion current density (*i*_corr_), polarization resistance (*R*_p_), corrosion rate (CR), the anodic (*β*a) and cathodic (*β*c) Tafel slopes.

**Table 6:** Electrochemical parameters derived from Tafel lines for the all samples under studies in 0.6 M NaCl at 25 °C.

Samples	*B*_a_ (V.dec)	*B*_c_ (V.dec)	*E*_corr_ (V) (Ag/AgCl)	*I*_corr_ (A.cm^−2^)	*R*_p_ Ω.cm^2^	C.R (mm.year^−1^)	± *SD*	*E* %
BD	0.148	0.172	− 0.610	5.82 × 10^−4^	59.2	6.76	6 × 10^–4^	–
P	0.244	0.229	− 0.585	3.19 × 10^–4^	160.5	3.72	1.5 × 10^–4^	44.9
S1	0.161	0.131	− 0.556	2.16 × 10^–4^	145.1	3.34	2 × 10^–4^	50.6
S2	0.113	0.151	− 0.608	1.03 × 10^–4^	272.4	2.39	1 × 10^–5^	64.6
S3	0.167	0.313	− 0.314	9.97 × 10^–6^	6921	0.15	1 × 10^–5^	97.7

BD is the most corroded sample, exhibiting a high corrosion current density of 5.82 × 10^−4^ A cm^−2^. P is less corroded than BD due to the presence of the native oxide, which delays the penetration of electrolytes to the surface. However, the natural patina does not cover the surface uniformly, and parts of this native oxide dissolve in the aggressive media, resulting in lower inhibition efficiency than the past coated fragments (S1, S2).

**Fig. 8:**
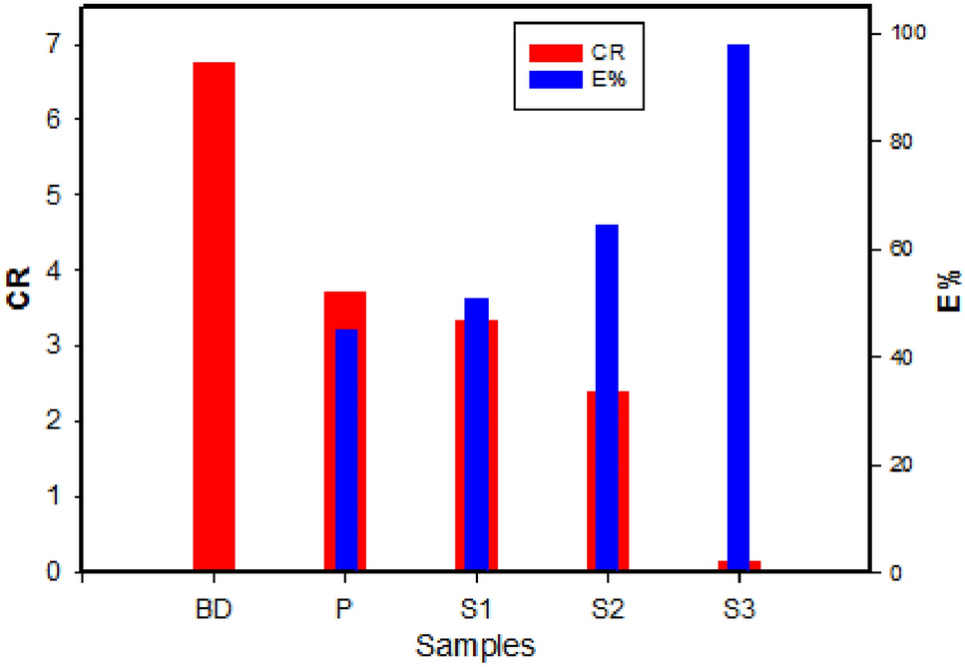
The variation of CR and the inhibition efficiency of different coin fragments in 0.6 M NaCl.

The protective properties of different fragments were compared in terms of decreasing corrosion rate and increasing efficiency. Figure 8 shows the relationship between corrosion rate (CR) and inhibition efficiency of different fragments under study. It is evident that as CR decreases, efficiency increases. Poor protective properties are observed in the bronze diseases BD sample. Patinated-freshly coated sample (S3) shows a significantly lower current density of 9.97 × 10^–6^ Acm^−2^ and a higher inhibition efficiency of 97.7%. This fresh coating acts as a blocking layer against corrosive media, indicating good protection.

#### Cyclic voltammetric measurement (CV)

**Fig. 9:**
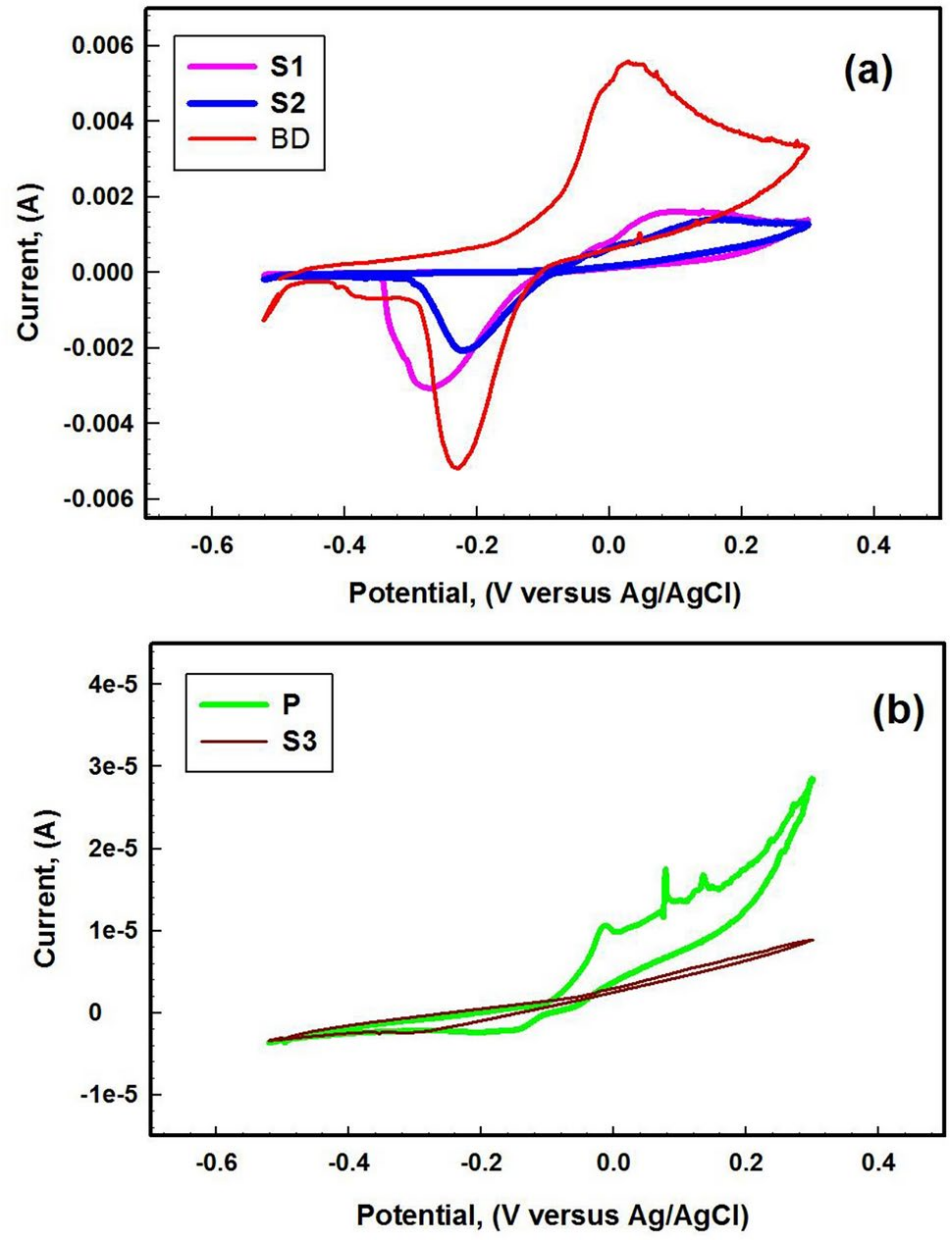
Cyclic voltammetry of studied coin fragments (**a**) BD, S1, andS2, (**b**) P and S3.

Cyclic voltammetry (CV) is a powerful analytical tool for investigating the electrochemical properties of materials, providing valuable insights into oxidation–reduction behavior^69,72^. Figure 9 reveals distinct differences in the shapes of the voltammograms. By comparing the current density values (Acm^−2^), it becomes evident that the freshly coated sample has lower current and greater stability. In Fig. 9a shows the CV curves of BD, S1, and S2. BD illustrates notable distinctions. BD displays high oxidation at 22 mV and reduction peaks at − 220 mV, contrasting with the oxidation peaks (68,123 mV) and reduction peaks (− 275, − 214 mV) observed for S1 and S2 respectively. In Fig. 9b depicts oxidation peaks (22, 81 and 134 mV) and reduction peaks (− 182 mV) for P.

S3, notably, did not exhibit an oxidation current, indicating a lack of peak in the voltammograms and suggesting the stability of the coating surface^55^. The oxidation peaks attributed to copper signify the formation of cuprous ions (Cu⁺) and the subsequent oxidation of cuprous ions (Cu⁺) to cupric ions (Cu^2^⁺). Shifts in the peak potentials can indicate changes in the electrochemical environment or the influence of surface treatments. Comparing the CV plots of freshly coated samples with past one allows us to understand the stability and effectiveness of the Paraloid B-72® coating. The presence of protective layers like Paraloid B-72® can be inferred from the decrease of anodic peak, signifying reduced oxidation of the bronze surface. A diminished intensity or absence of such peaks in the coated sample would indicate effective corrosion resistance.

The XRD spectra reveal that the primary component is cuprite Cu_2_O, and, through extension of the oxidation process, a thin solid CuO layer is formed on Cu_2_O^56,73^. In the P and S3 samples, the gradual coverage of the surface by copper oxide enhances the effective surface area for converting Cu(II) to Cu(I) as depicted^74^.

### The findings of this study

This study highlights the relationship between the chemical composition of archaeological coins and their corrosion behavior. SEM–EDX analyses offer valuable insights into the elemental composition and corrosion products. Corrosion tests further clarify the effectiveness of different treatments. Freshly applied Paraloid B-72® demonstrates superior barrier properties, notably reducing corrosion rates compared to untreated and long-term treated samples. However, its effectiveness diminishes over time, likely due to environmental exposure.

The correlation between chemical composition, especially the presence of chloride ions, and corrosion performance suggests that destabilization of the protective patina layer can accelerate corrosion. The comparative analysis underscores the necessity for continuous monitoring and reevaluation of conservation strategies to ensure the long-term effectiveness. The XRD analysis aligns with electrochemical measurements aiding interpretation of polarization and impedance study results. Understanding the composition of the corrosion products facilitates a clearer interpretation of the electrochemical behavior observed in polarization, cyclic voltammetry, and impedance studies. Comparing fresh and aged coatings highlights the durability and long-term protective properties of the treatment.

## Conclusion

Various bronze fragments, part of a collection of Greco-Roman coins were excavated from saline clay soil inside a pottery pot. A set of coin fragments was selected for analysis to determine their chemical composition, patina nature, and surface corrosion products using X-ray diffraction, stereo microscopy, and scanning electron microscopy with energy dispersive spectrometry.

This study confirms that the bronze materials of coin fragments, including copper, tin, and lead. The corrosion region were characterized by the presence of copper hydroxyl chloride [Cu_2_ (OH)_3_ Cl] and soil encrustations, with cuprite (Cu_2_O) as the primary compound on the patinated surface. The selected fragments included those affected by the bronze disease (BD), natural patina (P), two-year-past coated coin fragments (S1 and S2), and freshly coated fragments treated with 3% Paraloid B-72® (S3).

After removing the corrosion products, the protective efficiency of the fragments was evaluated using electrochemical techniques such as potentiodynamic polarization (PDP), electrochemical impedance spectroscopy (EIS), and cyclic voltammetry (CV). EIS results were consistent with the PDP findings.

Based on the results the patinated coin has a protective layer of native oxide similar to the previously coated one, while the most corroded fragment exhibits bronze disease. The freshly coated patinated fragment offers the highest level of protection. However, the efficiency of the previously coated coin is lower than that of the freshly coated one, attributed to coating failure and the color change to yellow one. Due to this limitation, maintaining the Paraloid B-72® coating is crucial to prevent corrosion during long-term storage under uncontrolled conditions. Based on the results, we recommend using the economically reversible and transparent Paraloid B-72® coating for only two years. This process involves carefully removing the old coating with suitable solvents and applying a fresh layer. To enhance adhesion and effectiveness, thoroughly clean the coin’s surface before reapplication.

## Supplementary Information

Supplementary Information.

## Data Availability

All data generated or analyzed during this study are included in this published article.
