# Supplementary material for: Evaluation of the corrosion resistance of bronze patina or/and protective coating on the surface of the archaeological coins
Source: Sci Rep. 2025 Jan 18;15:2361. doi: 10.1038/s41598-025-85290-x (PMC11742038; doi:10.1038/s41598-025-85290-x)
Supplement: Supplementary file 1 — Supplementary Information. [file 41598_2025_85290_MOESM1_ESM.docx]

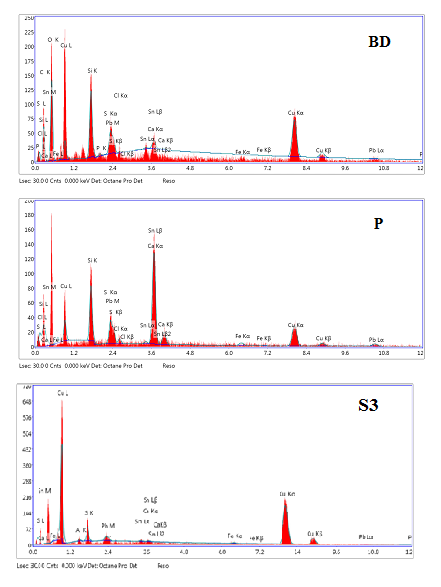


**Supplementary Figure 1**: EDX elemental analysis of bronze coins, (BD) coin suffer bronze disease, (P) coin naturally patinated , and (S3) coated coin by Paraloid B-72
